# Shoulder Dislocation Incidence and Risk Factors—Rural vs. Urban Populations of Poland

**DOI:** 10.3390/ijerph191911857

**Published:** 2022-09-20

**Authors:** Karol Szyluk, Paweł Niemiec, Dominik Sieroń, Dawid Lukoszek, Marcin Gierek, Andrzej Lorek, Andreas Christe

**Affiliations:** 1 Department of Physiotherapy, Faculty of Health Sciences in Katowice, Medical University of Silesia in Katowice, 40-752 Katowice, Poland; 2 District Hospital of Orthopaedics and Trauma Surgery, Bytomska 62 St., 41-940 Piekary Śląskie, Poland; 3 Department of Biochemistry and Medical Genetics, Faculty of Health Sciences in Katowice, Medical University of Silesia in Katowice, 40-752 Katowice, Poland; 4 Department of Radiology SLS, Inselspital, Bern University Hospital, University of Bern, Freiburgstrasse 10, 3010 Bern, Switzerland; 5 Dawid Lukoszek Physiotherapy Osteopathy, 42-690 Hanusek, Poland; 6 Center for Burns Treatment im. Dr Sakiel, ul. Jana Pawła II 2, 41-100 Siemianowice Śląskie, Poland; 7 Department of Oncological Surgery, Prof. Kornel Gibiński Independent Public Central Clinical Hospital, Medical University of Silesia in Katowice, 40-514 Katowice, Poland

**Keywords:** shoulder, dislocation, epidemiology, incidence, risk factors, rural, urban

## Abstract

(1) Background: The aim of this study was to analyze the incidence of shoulder dislocation and to estimate non-modifiable risk factors in rural and urban subgroups in Poland. (2) Methods: The study covered the entire Polish population, divided into urban and rural subgroups and observed between 1 January 2014 and 31 December 2014. The study population consisted of Polish patients with a diagnosis of shoulder dislocation (S43.0) in accordance with the International Statistical Classification of Diseases and Related Health Problems (ICD-10). Records were obtained from the public health care provider National Health Found (Narodowy Fundusz Zdrowia, NFZ). Based on these data, we assessed shoulder dislocation incidence and risk rates, stratifying the study sample by sex, age and place of residence (rural or urban) using the Central Statistical Office (GUS) personal territorial code (TERYT). (3) The incidence was 25.97/100,000 person-years in rural areas and 25.62/100,000 person-years in urban areas. We did not find significant differences in the incidence between the two subgroups. The highest incidence (75.12/100,000 person-years) and the highest risk for shoulder dislocation were found among subjects 80+ years old living in urban areas. Furthermore, men in the third decade of their life living in urban areas showed the highest risk (OR = 7.8, 95% CI; 6.44–9.45, *p* < 0.001). In both subgroups, the likelihood of shoulder dislocation was significantly lower for the female sex and among children ≤9 years old. However, girls living in rural areas presented with a significantly higher likelihood for dislocation compared with their peers living in urban environments. (4) Conclusions: No significant difference in the incidence rate of shoulder dislocation between Polish residents living in rural and urban areas emerged. The highest incidence was observed among female subjects 80+ years old living in urban environments. The highest risk was found among men in the third decade of their life living in urban areas. In addition, girls in the first decade of their life living in rural areas had more shoulder dislocations than girls living in urban environments. Shoulder dislocation is dominant in female subjects aged 70–79 living in rural areas and in females 80+ years old living in urban areas.

## 1. Introduction

Shoulder dislocation is a trauma that nearly all emergency department doctors confront at some point during their professional careers due to its high incidence. In Poland, the incidence of shoulder dislocation is 26.69/100,000 person-years [1]. Based on the data available in the literature, incidence rates range from 8.2 to 26.2/100,000 person-years [2,3]. Despite discrepancies in the data, there is consensus that the shoulder joint is the most frequently dislocated joint in the human skeletal system [2,4,5,6]. Considering modifiable risk factors, athletes are one of the most vulnerable subgroups, in particular athletes practicing contact sports and sports that require the arms moving to an overhead position [7,8].

Of all joints in the human body, the shoulder joint is the most mobile, due to its unique anatomical structure: the articular surface of the humeral head is indeed three times larger than the articular surface of the acetabulum, allowing for a wider range of movement [9]. Joint stabilizers (both static and dynamic) further impact the joint’s mobility, affecting the entire upper limb’s ability to grasp and manipulate objects with adequate efficiency [10]. On the other side, this high mobility is also responsible for the increased predisposition to dislocations.

Epidemiological studies are missing on shoulder dislocations based on metadata analyses describing or comparing entire populations or their significant subgroups beyond risk groups. Our aim was to start addressing this gap by comparing the incidence of shoulder dislocation in rural and urban subgroups in Poland, which also allowed for an indirect assessment of the existing non-modifiable risk factors in both subgroups as well as a first evaluation of the correlation between place of residence and health care availability.

## 2. Materials and Methods

### 2.1. Identification of the Test Groups

The National Health Fund (NFZ) keeps a register of all medical diagnoses coded in accordance with the International Statistical Classification of Diseases and Related Health Problems (ICD-10) throughout Poland. We screened the data collected by this fund between 1 January 2014 and 31 December 2014 to identify patients with posttraumatic dislocation of the shoulder using ICD-10 code S43.0 (dislocation of the shoulder joint). Only four-digit codes precisely related to the specified diagnosis were considered. Each patient with a dislocation was given a unique numerical identifier, for reasons of anonymity and for the protection of personal data.

### 2.2. Population Data

As the reference group, we considered the entire population of Poland not suffering shoulder dislocation in 2014 (from 1 January 2014 to 31 December 2014). Basic population data such as number of inhabitants per town and village using territorial code (TERYT), age and sex were obtained from the Central Statistical Office (GUS), the national agency for collecting population data, including demographics, in Poland [11].

### 2.3. Inclusion and Exclusion Criteria for the Test Group (Patients with Shoulder Dislocation)

#### 2.3.1. Inclusion Criteria

- Polish residents as verified by their territorial code;
- One-off dislocations of the shoulder coded during the studied period;
- Diagnoses performed in emergency rooms and coded according to the ICD-10 classification.

#### 2.3.2. Exclusion Criteria

- Shoulder dislocation diagnoses not coded in accordance with ICD-10;
- Consecutive shoulder dislocations for the same patient, considered recurrences;
- Patients not residing in Poland.

Based on the above criteria, we obtained a group of patients who reported to an emergency department with a single posttraumatic shoulder dislocation diagnosis during the period under consideration.

### 2.4. Determination of the Incidence and Risk Rates for Shoulder Dislocation

Using the NFZ data, in which each S43.0 diagnosis was accompanied by a territorial code, the study group was divided into two subgroups: rural and urban. Patients were further stratified based on age and sex. The demographic data were analyzed, and for each of the outlined groups, as well as for the entire study population, the number and frequency of posttraumatic shoulder dislocations over the studied period were determined. The incidence rate was expressed as the number of new registered cases per 100,000 person-years. These results were used to compare the relative incidence rates in rural and urban subgroups both as a whole and stratified for age and sex. To estimate the risk of shoulder dislocation, we calculated the odds ratios (ORs) of urban vs. rural shoulder dislocation for the entire population and for the age and sex subgroups as well as the risk of dislocation in rural vs. urban environments.

### 2.5. Statistical Analysis

Microsoft Excel software v2002 (compilation 1252.22215) (Microsoft Corporation, Redmond, WA, USA) was used to store data and generate charts. Differences in the frequency of shoulder dislocation in regard to gender and age ranges were assessed with Pearson’s chi-square (χ^2^) test. Odds ratios (OR) with 95% confidence intervals (CI) were calculated using Epi Info™ 7.2 software, developed by the Centers for Disease Control and Prevention (Atlanta, GA, USA). Statistica 12.0 software (STATSOFT, Tulsa, OK, USA) was used in all of the above analyses, and *p*-values less than 0.05 were considered to be statistically significant.

## 3. Results

The population of Poland for the observed period amounted to 38,478,602 individuals, among whom, 15,262,250 (39.67%) resided in rural areas, with a female population of 7,652,607 (50.14%) and a male population of 7,609,643 (49.86%), while the remaining 23,216,352 (60.33%) people resided in urban areas, with a female population of 12,206,186 (52.58%) and a male population of 11,010,166 (47.42%).

### 3.1. Analysis of the Number of Shoulder Dislocations

**Figure 1:**
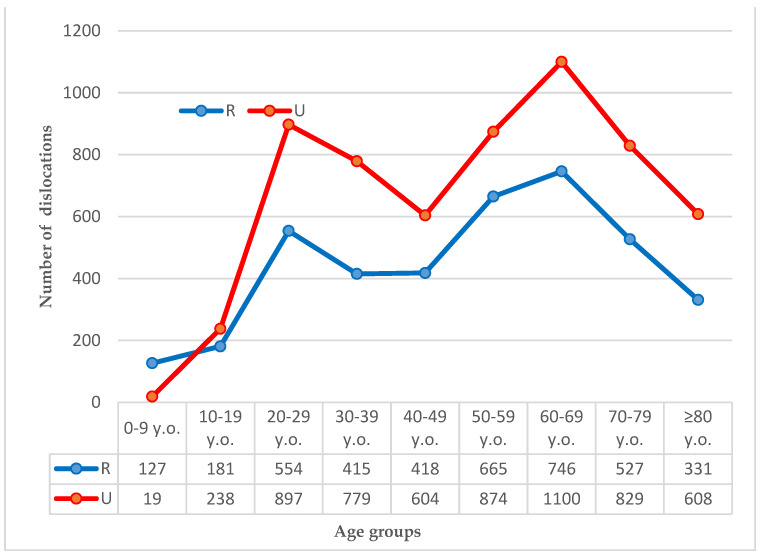
Number of dislocations per age group. Legend: R, rural population; U, urban population.

During the study period, 9912 shoulder dislocations were registered: 3964 (39.99%) dislocations took place in rural areas, of which 2602 (65.64%) occurred in men and 1362 (34.36%) in women. The other 5948 (60.01%) dislocations were reported in urban areas, including 3745 (62.96%) dislocations occurring in men and 2203 (37.04%) in women. The highest number of dislocations was reported in patients 60 to 69 years old both in rural and urban subgroups, with 746 and 1100 dislocations respectively. The details are presented in Figure 1.

**Figure 2:**
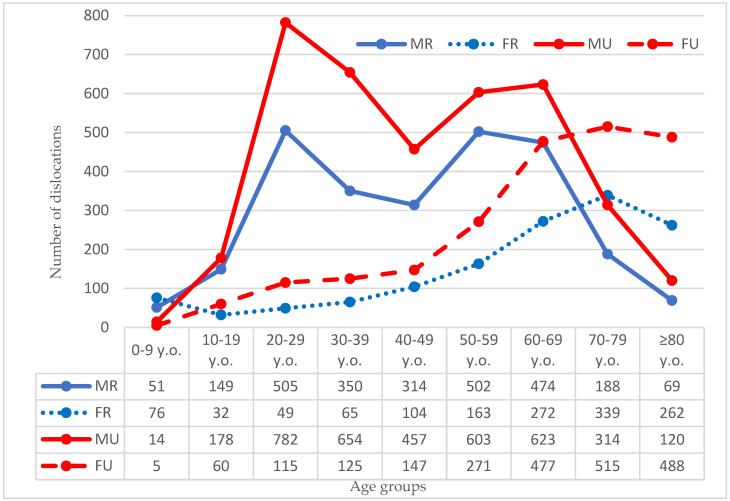
Number of dislocations by age and sex. Legend: MR, male rural population; FR female rural population; MU, male urban population; FU, female urban population.

Stratifying the groups by age and sex, the largest total number of incidents was found in men in their third decade of life, both in the countryside and in the city, with 505 and 782 dislocations, respectively (Figure 2).

**Figure 3:**
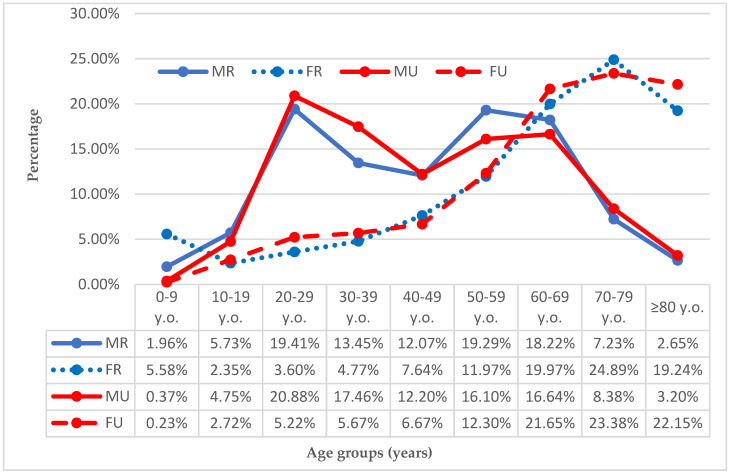
Prevalence (%) of dislocations by age and sex. Legend: FR, female rural population; FU, female urban population; MR, male rural population; MU, male urban population.

For the female population, the highest prevalence of dislocations was found in the 60–69 age subgroup living in rural areas (24.89%). For the male population, the 20–29 age subgroup residing in urban areas presented the highest prevalence (20.88%) of dislocations. The dislocation prevalences for all age subgroups analyzed are reported in Figure 3.

### 3.2. Incidence Rates in Urban and Rural Subgroups

**Figure 4:**
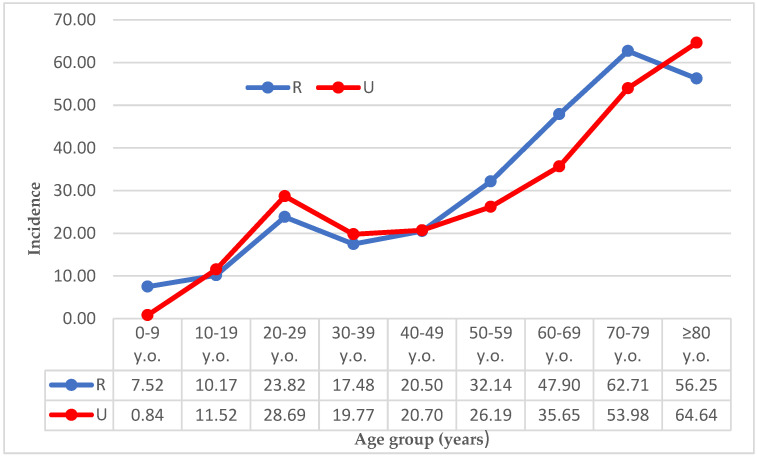
Incidence rate (nr. of cases/100,000 person-years) of dislocations by age group in rural and urban populations. Legend: R, rural population; U, urban population.

The incidence rate was 25.97/100,000 person-years in rural areas and 25.62/100,000 person-years in urban areas. The highest incidence rate was found in people older than 80 (64.64/100,000 person-years) living in urban environments, while in rural areas, the 70–79 age group presented the highest rate of dislocations (62.71/100,000 person-years). A comparison of the incidence rates in rural and urban subgroups is shown in Figure 4.

**Figure 5:**
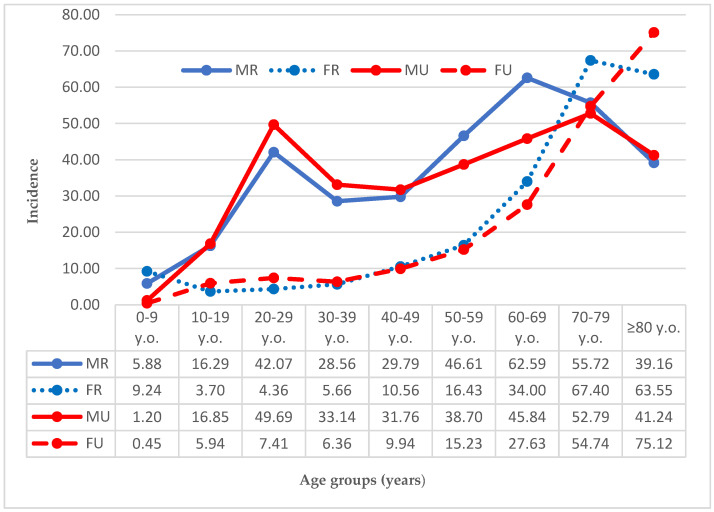
Incidence rate (nr. of cases/100,000 person-years) of dislocations by age and sex. Legend: MR, male rural population male; FR, female rural population; MU, male urban population; FU, female urban population.

Taking into account age and sex, the highest incidence rate was found in female city residents 80+ years old (75.12/100,000 person-years). Among men, the highest incidence rate was found in the 60–69 age group living in rural areas (62.59/100,000 person-years). Incidence rates by sex are presented in Figure 5.

No significant correlation (*p* = 0.505) between the number of dislocations and the place of residence emerged from the analysis. However, the number of dislocations correlated significantly with age (*p* < 0.001) and sex (*p* < 0.001).

### 3.3. Risk Assessment of Shoulder Dislocation Depending on the Place of Residence

The analysis of the odds ratios for the rural and urban subgroups independently (Table 1 and Table 2) showed that the lowest expected risk of dislocation was in the 0–9 age subgroup of the female population, especially among girls living in urban environments. The reference group consisted of the whole female population (female residents: OR = 0.02, 95% CI; 0.01–0.05, *p* < 0.001). The risk of dislocation started to increase in the sixth (rural population) and seventh (urban population) decades of life and continued to increase in the oldest age groups. For both subgroups, the highest risk of dislocation was among the two oldest age groups. The highest risk of dislocation was recorded in women in the 80+ age group in urban areas and the 70–79 age group in rural areas. In the rural population, the highest risk for men in the rural population was found in the age ranges 60–79 years, and it was in the age ranges of 20–29 and 70–79 for men living in urban areas. Odds ratios for both subgroups are detailed below (Table 1, rural population; Table 2, urban population).

### 3.4. Dislocation Risk in Rural vs. Urban Subgroups

The comparison of the risk (OR) for the first event of shoulder dislocation among urban and rural subgroups, taking into account the stratification by age and sex, unraveled that girls aged 0–9 years living in the countryside are the most exposed to shoulder dislocation accidents. Furthermore, the risk of dislocation was shown to progressively increase in both sexes between the ages of 50 and 80 in rural and urban subgroups, with the exception of women in their sixth decade of life. The comparison of the age-related odds ratios (ORs) for the first shoulder dislocation event within rural and urban subgroups is presented in Table 3.

### 3.5. Risk Assessment of Shoulder Dislocation Depending on Place of Residence and Sex

There is an increased risk for the occurrence of the first shoulder dislocation events in men of both subgroups (urban areas: OR = 1.7, 95% CI; 1.61–1.79, *p* < 0.001; rural areas: OR = 1.92, 95% CI; 1.8–2.05, *p* < 0.001). The comparison of the likelihood for dislocation taking into account sex and age group showed that men in their 20s living in urban areas share the highest risk (OR = 7.8, 95% CI; 6.44–9.45, *p* < 0.001). Moreover, a significantly high risk was found also in the subgroup of men aged 30–60 years old living in rural areas (see Table 4).

## 4. Discussion

In this study, the incidence rates of shoulder joint dislocation were 25.97/100,000 person-years in the rural subgroup, and 25.62/100,000 person-years in the urban subgroup. The highest incidence rate (75.12/100,000 person-years) was found among women aged 80+ years old living in urban areas, while women 60–69 years old living in rural areas shared the highest prevalence of shoulder dislocations within the female population. On the other hand, while the lowest event rate was found among girls aged 0–9 years old for both rural and urban subgroups, a significantly higher risk for dislocation associated with living in rural areas emerged from the differential comparison based on place of residence.

The main results of this study are in line with those obtained previously from the analysis of metadata of the Polish population that did not take into consideration the place of residence (26.69/ 100,000 person-years) [1], as well as with data presented by other authors, e.g., Zacchilli (USA) (23.9/100,000 person-years) and Leroux (Canada) 23.1/100,000 person-years [3,12]. Both authors did not investigate the dislocation incidence in correlation with the patients’ age or the risk extent.

No statistically significant differences emerged from the analysis of the incidence rates for urban and rural subgroups. The incidence rates in the male and female populations living in rural areas were 34.19/100,000 person-years and 17.8/100,000 person-years respectively. Similar results were recorded for both sexes in the urban population (34.01/100,000 person-years and 18.05/100,000 person-years, respectively, for male and female subgroups). The incidence rates for both genders was similar to those reported in epidemiological studies from other authors: Liaavag et al., reported incidence rates of 34.8/100,000 person-years in the male population and 17.9/100,000 person-years in the female population [13], while Zacchilli et al., found incidence rates of 34.9/100,000 person-years for men and 13.3/100,000 person-years for women [3].

Significantly higher incidence rates were found in studies of risk groups and/or performed on small populations. For soldiers, Amako et al., reported an incidence rate of 410/100,000 person-years [14], and Kardouni et al., a rate of 313/100,000 person-years [15]. In another study, the frequency of shoulder dislocation in players practicing rugby was 100/100,000 person-years in men, and 50/100,000 person-years in women [16]. Recent publications (2020–2022) presented data from studies of rugby players, American football players, and a group of children and teenagers [17,18,19,20,21]. These incidence rates are peculiar to these specific subgroups and not representative of the general population. Studies conducted on small risk groups bring extremely important and useful information about these subgroups of people, though the results cannot be extrapolated to the general population, and their predictive value for other/larger groups is limited.

Unfortunately, there is a lack of epidemiological studies on wider, heterogeneous groups, e.g., large populations living in urban and rural environments, and studies comparing data from such subgroups. In 1984, Simonet et al., for the first time analyzed the incidence of shoulder dislocation in urban and rural populations using place of residence as a criterion for comparison. The study object was the population of Olmsted, Minnesota. The incidence in the city amounted to 7.6/100,000 person-years in urban areas and 10.2/100,000 person-years in rural areas [22]. We attribute the differences between these numbers and ours to the different study group sizes. Simonet analyzed 116 patients with shoulder dislocation, while our results are derived from the analysis of 9912 dislocations overall. Moreover, the study By Simonet et al., was performed in the 1970s on a population of 880,000 residents, while our subjects were the entire Polish population (38,478,602 people) [22]. Despite these differences, Simonet et al., as in our case, did not find statistically significant differences between incidence rates in rural and urban areas [22].

Shields et al., show relatively up-to-date data on the urban population of the UK, for which an incidence rate of 21.9/100,000 person-years is reported [23]. Shields et al., report a bimodal incidence peak for men aged 15–24 (421/100,000 person-years) and 85+ (509/100,000 person-years) and a peak incidence of 457/100,000 person-years in men aged 65–74 [23]. In the present study, a similar bimodal/unimodal incidence peaks trend was observed. However, peak incidences were found in different age ranges for our Polish urban subgroups. Peak incidence rates for men were found at ages 20–29 and 70–79 (49.69/100,000 person-years and 52.79/100,000 person-years respectively), while for women, the peak was unimodal with 75.12/100,000 person-years at 80+ years of age. In our opinion, the reasons for these differences lay in the inclusion criteria for the study—our study examines the entire population, while Shields only includes inhabitants over 15 years of age. Moreover, the differences presented above may be influenced by the higher life expectancy of men in the UK [23].

An analysis of groups without age and sex stratification showed that the place of residence (urban vs. rural) was not a risk factor for the first event of shoulder dislocation (OR = 1.01, 95% CI; 0.97–1.05; *p* = 0.043; *p* = 0.504). With our analysis of the risk of shoulder dislocation based on sex and pre-defined age groups, we could identify differences in the risk distribution among groups. The highest risk was found for men between 20 and 29 years old living in urban areas (OR = 7.8, 95% CI; 6.44–9.45, *p* < 0.001). For the age range 30–60 years, the highest risk is in men living in rural areas. This is in agreement with other data available from the medical literature. We think that the higher risk of dislocation in this subgroup is due to the significant exposure to trauma associated with manual labor on a farm, but confirming this assumption would require a detailed study of a smaller population. Other authors also reported that in the urban population, the increased risk of dislocation among young men is a consequence of their frequent participation in sports [3,22,24,25,26]. However, we want to emphasize that due to lack of comparable studies, the conclusions above require further verification. Similar results were obtained in the male (OR = 1.01, 95% CI; 0.96–1.06, *p* = 0.836) and female (OR = 0.99, 95% CI; 0.93–1.06, *p* = 0.685) subpopulations.

Our analysis of the non-modifiable risk factors demonstrated the following: the male gender represents a risk factor for shoulder dislocation both in the rural (OR = 1.92, 95% CI; 1.8–2.05, *p* < 0.001) and urban (OR = 1, 7, 95%CI; 1,61–1.79, *p* < 0.001) subpopulations. These results are consistent with other data available in the literature on the topic. Leroux et al., showed a 6.7 times greater risk of primary shoulder dislocation in men under 20 years old (98.3/100,000 person-years) than in women belonging to the same age group (*p* < 0.001) [12]. In the study by Tas et al., the incidence of shoulder dislocation in men was 8.0/100,000 person-years, while in women, it was 2.58/100,000 person-years [27]. Liavaag et al., found 82.2/100,000 person-years (95% CI; 71.7–92.8) in men and 30.9/100,000 person-years (95% CI; 24.5–37.3) in women [13]. The male gender represents a risk factor for the recurrence of shoulder dislocation in the 20–29 age group (OR = 2.59, 95% CI 2.38–2.83, *p* < 10^−10^) [28].

In summary, the highest risk for shoulder dislocation in the rural subpopulation was found in the 70–79 age group (OR = 4.17, 95% CI; 4.17–5.33, *p* < 0.001), while in the urban population, it was shared by women aged 80+ (5.07, 95% CI; 4.58–5.61, *p* < 0.001). While the propensity to injuries in older patients can be attributed to many factors (degenerative changes, balance disorders, decreased physical fitness), the high exposure we found in girls younger than 9 years old should prompt us to consider the introduction of both prophylactic measures against injuries at an early age as well as educational measures to promote awareness in adult caretakers. However, it should be emphasized that in both urban and rural subpopulations, belonging to the female gender and being in the age range 0–9 years old are both protective factors even though living in rural areas implies a 20-fold higher risk of dislocation than living in urban areas, which should raise some concern. According to the authors of this paper, this situation might result from the specific activities and lifestyle in rural areas: the living and working place may entail more risks, which both children and their caretakers may fail to recognize. According to epidemiological data, the incidence of injuries in children aged 0–9 living in the countryside is indeed strictly related to living and working in this specific environment (animals, agricultural machines). 

The risk groups identified here may differ in terms of traumatic complications, diagnosis and treatment. Therefore, doctors working at emergency rooms should have basic skills consistent with the available knowledge on the epidemiology and management of patient with a dislocated shoulder in different age groups. 

Our study is the first countrywide study as we analyze data of the entire Polish population, without exclusions based on, e.g., age or gender. The heterogeneity of such a large group averages out the real incidence of shoulder dislocation accidents in found in specific risk groups (like rugby players) and provides a good approximation of the risk for the general population. However, only non-modifiable factors like sex and gender were available in the NFZ database used for this study. The inability to identify the causes of dislocation and the patients’ medical history (additional diseases, dislocations, past injuries, consequences of dislocations, applied treatment BMI, level of sports activity or type of work, etc.) accounts for some limitations and reduces the predictive power of these results for other populations/subgroups that might benefit from a specific, smaller group analysis instead.

In the future, it will be important to refine the analysis and provide more precise data as most emergency doctors will encounter shoulder dislocations. We believe that further population studies are necessary for gaining knowledge about the impacts of both non-modifiable risk factors and modifiable risk factors (like level of sports activity or type of work performed) on dislocations. Further studies should also provide information on the risk of recurrence and development of shoulder instability for all subgroups studied, which will complement the current knowledge in this field. We also believe that research based on smaller populations of towns and villages is necessary and will allow for a more fine-tuned representation of the incidence and risk distribution. The subgroup of girls aged 0–9 years living in rural areas also calls for more specific analyses to explain the significantly higher risk of dislocation observed here.

## 5. Conclusions

Based on our data, no differences in the incidence rates of dislocations in urban and rural subgroups can be identified, with the exception of girls in their first life decade. 

On the other hand, the most important risk factor is represented by age and gender. In females, the highest incidence rate is found in the 70–79 age group in rural areas and in the 80+ age group in urban areas. Female gender and age in the first life decade are strong protective factors within both studied subpopulations, but girls aged 0–9 living in rural areas have a significantly higher risk of shoulder dislocation than those living in urban areas. Considering the entire Polish population, men in their third life decade have an almost eight times higher risk of shoulder dislocation than the rest of the population taken together.

## Figures and Tables

**Table 1:** Odds ratios (ORs) for the first shoulder dislocation event in residents living in rural areas.

Age Range (Years)	Group		Number of Events (*n*)	Population at Risk (*n*)	OR	95%CI	*p*
**0–9**	*entire group*	0–9	127	1,689,642	0.27	0.23–0.32	<0.001
		vs. others	3837	13,572,608			
	*males*	0–9	51	867,073	0.16	0.12–0.21	<0.001
		vs. others	2551	6,742,570			
	*females*	0–9	76	822,569	0.49	0.39–0.62	<0.001
		vs. others	1286	6,830,038			
**10–19**	*entire group*	10–19	181	1,779,092	0.36	0.31–0.42	<0.001
		vs. others	3783	13,483,158			
	*males*	10–19	149	914,493	0.44	0.37–0.52	<0.001
		vs. others	2453	6,695,150			
	*females*	10–19	32	864,599	0.19	0.13–0.27	<0.001
		vs. others	1330	6,788,008			
**20–29**	*entire group*	20–29	554	2,324,794	0.9	0.82–0.98	0.028
		vs. others	3410	12,937,456			
	*males*	20–29	505	1,199,937	1.29	1.17–1.42	<0.001
		vs. others	2097	6,409,706			
	*females*	20–29	49	1,124,857	0.22	0.17–0.29	<0.001
		vs. others	1313	6,527,750			
**30–39**	*entire group*	30–39	415	2,373,672	0.63	0.57–0.7	<0.001
		vs. others	3549	12,888,578			
	*males*	30–39	350	1,225,090	0.81	0.72–0.91	<0.001
		vs. others	2252	6,384,553			
	*females*	30–39	65	1,148,582	0.28	0.22–0.36	<0.001
		vs. others	1297	6,504,025			
**40–49**	*entire group*	40–49	418	2,038,318	0.76	0.69–0.84	<0.001
		vs. others	3546	13,223,932			
	*males*	40–49	314	1,053,778	0.85	0.76–0.96	0.009
		vs. others	2288	6,555,865			
	*females*	40–49	104	984,540	0.56	0.46–0.68	<0.001
		vs. others	1258	6,668,067			
**50–59**	*entire group*	50–59	665	2,068,286	1.29	1.19–1.4	<0.001
		vs. others	3299	13,193,964			
	*males*	50–59	502	1,076,546	1.45	1.32–1.6	<0.001
		vs. others	2100	6,533,097			
	*females*	50–59	163	991,740	0.91	0.77–1.07	0.276
		vs. others	1199	6,660,867			
**60–69**	*entire group*	60–69	746	1,556,547	2.04	1.88–2.21	<0.001
		vs. others	3218	13,705,703			
	*males*	60–69	474	756,785	2.02	1.83–2.23	<0.001
		vs. others	2128	6,852,858			
	*females*	60–69	272	799,762	2.14	1.87–2.44	<0.001
		vs. others	1090	6,852,845			
**70–79**	*entire group*	70–79	527	839,820	2.63	2.4–2.88	<0.001
		vs. others	3437	14,422,430			
	*males*	70–79	188	337,209	1.68	1.45–1.95	<0.001
		vs. others	2414	7,272,434			
	*females*	70–79	339	502,611	4.71	4.17–5.33	<0.001
		vs. others	1023	7,149,996			
**80+**	*entire group*	80+	331	588,115	2.27	2.03–2.54	<0.001
		vs. others	3633	14,674,135			
	*males*	80+	69	176,130	1.15	0.91–1.46	0.253
		vs. others	2533	7,433,513			
	*females*	80+	262	411,985	4.19	3.66–4.79	<0.001
		vs. others	1100	7,240,622			

Legend: CI, confidence interval; *n*, number of events; OR, odds ratio; *p*, statistical significance.

**Table 2:** Odds ratios (OR) for the first shoulder dislocation event in residents living in urban areas.

Age Range	Group		Number of Events (*n*)	Population at Risk (*n*)	OR	95%CI	*p*
**0–9**	*entire group*	0–9	19	2,267,722	0.03	0.02–0.05	<0.001
		vs. others	5929	20,948,630			
	*males*	0–9	14	1,164,278	0.03	0.02–0.05	<0.001
		vs. others	3731	9,845,888			
	*females*	0–9	5	1,103,444	0.02	0.01–0.05	<0.001
		vs. others	2198	11,102,742			
**10–19**	*entire group*	10–19	238	2,065,627	0.43	0.38–0.49	<0.001
		vs. others	5710	21,150,725			
	*males*	10–19	178	1,055,945	0.47	0.4–0.55	<0.001
		vs. others	3567	9,954,221			
	*females*	10–19	60	1,009,682	0.31	0.24–0.4	<0.001
		vs. others	2143	11,196,504			
**20–29**	*entire group*	20–29	897	3,125,132	1.14	1.06–1.22	<0.001
		vs. others	5051	20,091,220			
	*males*	20–29	782	1,573,032	1.58	1.46–1.71	<0.001
		vs. others	2963	9,437,134			
	*females*	20–29	115	1,552,100	0.38	0.31–0.46	<0.001
		vs. others	2088	10,654,086			
**30–39**	*entire group*	30–39	779	3,939,605	0.74	0.69–0.8	<0.001
		vs. others	5169	19,276,747			
	*males*	30–39	654	1,973,040	0.97	0.89–1.06	0.466
		vs. others	3091	9,037,126			
	*females*	30–39	125	1,966,565	0.31	0.26–0.37	<0.001
		vs. others	2078	10,239,621			
**40–49**	*entire group*	40–49	604	2,916,665	0.79	0.73–0.86	<0.001
		vs. others	5344	20,299,687			
	*males*	40–49	457	1,438,641	0.92	0.83–1.01	0.117
		vs. others	3288	9,571,525			
	*females*	40–49	147	1,478,024	0.52	0.44–0.61	<0.001
		vs. others	2056	10,728,162			
**50–59**	*entire group*	50–59	874	3,336,495	1.03	0.96–1.11	0.478
		vs. others	5074	19,879,857			
	*males*	50–59	603	1,557,639	1.16	1.06–1.27	0.001
		vs. others	3142	9,452,527			
	*females*	50–59	271	1,778,856	0.82	0.72–0.93	0.003
		vs. others	1932	10,427,330			
**60–69**	*entire group*	60–69	1100	3,084,428	1.48	1.39–1.58	<0.001
		vs. others	4848	20,131,924			
	*males*	60–69	623	1,358,551	1.42	1.3–1.55	<0.001
		vs. others	3122	9,651,615			
	*females*	60–69	477	1,725,877	1.68	1.52–1.86	<0.001
		vs. others	1726	10,480,309			
**70–79**	*entire group*	70–79	829	1,534,788	2.29	2.13–2.46	<0.001
		vs. others	5119	21,681,564			
	*males*	70–79	314	594,457	1.6	1.43–1.80	<0.001
		vs. others	3431	10,415,709			
	*females*	70–79	515	940,331	3.66	3.32–4.04	<0.001
		vs. others	1688	11,265,855			
**80+**	*entire group*	80+	608	939,942	2.7	2.48–2.94	<0.001
		vs. others	5340	22,276,410			
	*males*	80+	120	290,838	1.22	1.02–1.46	0.032
		vs. others	3625	10,719,328			
	*females*	80+	488	649,104	5.07	4.58–5.61	<0.001
		vs. others	1715	11,557,082			

Legend: CI, confidence interval; *n*, number of events; OR, odds ratio; *p*, statistical significance.

**Table 3:** Age-related odds ratios (ORs) for the first shoulder dislocation event in rural versus urban subgroups.

Age Range (Years)	Group		Number of Events (*n*)	Population at Risk (*n*)	OR	95% CI	*p*
**0–9**	*entire group*	rural	127	1,689,642	8.97	5.54–14.53	<0.001
		vs. urban	19	2,267,722			
	*males*	rural	51	867,073	4.89	2.71–8.83	<0.001
		vs. urban	14	1,164,278			
	*females*	rural	76	822,569	20.39	8.25–50.4	<0.001
		vs. urban	5	1,103,444			
**10–19**	*entire group*	rural	181	1,779,092	0.88	0.73–1.07	0.207
		vs. urban	238	2,065,627			
	*males*	rural	149	914,493	0.97	0.78–1.21	0.759
		vs. urban	178	1,055,945			
	*females*	rural	32	864,599	0.62	0.4–0.95	0.029
		vs. urban	60	1,009,682			
**20–29**	*entire group*	rural	554	2,324,794	0.83	0.75–0.92	0.001
		vs. urban	897	3,125,132			
	*males*	rural	505	1,199,937	0.85	0.76–0.95	0.003
		vs. urban	782	1,573,032			
	*females*	rural	49	1,124,857	0.59	0.42–0.82	0.002
		vs. urban	115	1,552,100			
**30–39**	*entire group*	rural	415	2,373,672	0.88	0.78–0.99	0.043
		vs. urban	779	3,939,605			
	*males*	rural	350	1,225,090	0.86	0.76–0.98	0.025
		vs. urban	654	1,973,040			
	*females*	rural	65	1,148,582	0.89	0.66–1.2	0.447
		vs. urban	125	1,966,565			
**40–49**	*entire group*	rural	418	2,038,318	0.99	0.87–1.12	0.878
		vs. urban	604	2,916,665			
	*males*	rural	314	1,053,778	0.94	0.81–1.09	0.383
		vs. urban	457	1,438,641			
	*females*	rural	104	984,540	1.06	0.82–1.36	0.638
		vs. urban	147	1,478,024			
**50–59**	*entire group*	rural	665	2,068,286	1.23	1.11–1.36	<0.001
		vs. urban	874	3,336,495			
	*males*	rural	502	1,076,546	1.2	1.07–1.35	0.002
		vs. urban	603	1,557,639			
	*females*	rural	163	991,740	1.08	0.89–1.31	0.444
		vs. urban	271	1,778,856			
**60–69**	*entire group*	rural	746	1,556,547	1.34	1.22–1.47	<0.001
		vs. urban	1100	3,084,428			
	*males*	rural	474	756,785	1.37	1.22–1.54	<0.001
		vs. urban	623	1,358,551			
	*females*	rural	272	799,762	1.23	1.06–1.43	0.006
		vs. urban	477	1,725,877			
**70–79**	*entire group*	rural	527	839,820	1.16	1.04–1.29	0.007
		vs. urban	829	1,534,788			
	*males*	rural	188	337,209	1.06	0.88–1.27	0.558
		vs. urban	314	594,457			
	*females*	rural	339	502,611	1.23	1.07–1.41	0.003
		vs. urban	515	940,331			
**80+**	*entire group*	rural	331	588,115	0.87	0.76–0.99	0.042
		vs. urban	608	939,942			
	*males*	rural	69	176,130	0.95	0.71–1.28	0.732
		vs. urban	120	290,838			
	*females*	rural	262	411,985	0.85	0.73–0.99	0.029
		vs. urban	488	649,104			

Legend: CI, confidence interval; *n*, number of events; OR, odds ratio; *p*, statistical significance.

**Table 4:** Comparison of shoulder dislocation risk based on sex and place of residence.

Age Range	Group		Number of Events (*n*)	Population at Risk (*n*)	OR	95% CI	*p*
**0–9**	*rural*	male	56	864,483	0.68	0.48–0.96	0.027
		vs. female	78	819,910			
	*urban*	male	13	1,148,952	1.76	0.7–4.41	0.221
		vs. female	7	1,090,347			
	*entire group*	male	69	2,013,435	0.77	0.56–1.06	0.106
		vs. female	85	1,910,257			
**10–19**	*rural*	male	145	975,900	2.7	1.96–3.71	<0.001
		vs. female	51	925,608			
	*urban*	male	213	1,118,561	3.09	2.34–4.07	<0.001
		vs. female	66	1,070,886			
	*entire group*	male	358	2,094,461	2.92	2.37–3.6	<0.001
		vs. female	117	1,996,494			
**20–29**	*rural*	male	503	1,234,219	7.02	5.44–9.06	<0.001
		vs. female	67	1,154,346			
	*urban*	male	923	1,741,559	7.8	6.44–9.45	<0.001
		vs. female	117	1,722,461			
	*entire group*	male	1426	2,975,778	7.49	6.42–8.73	<0.001
		vs. female	184	2,876,807			
**30–39**	*rural*	male	368	1,193,176	4.66	3.63–5.98	<0.001
		vs. female	74	1,117,146			
	*urban*	male	675	1,908,931	4.23	3.56–5.03	<0.001
		vs. female	159	1,902,980			
	*entire group*	male	1043	3,102,107	4.36	3.78–5.03	<0.001
		vs. female	233	3,020,126			
**40–49**	*rural*	male	355	1,034,686	3.69	2.92–4.66	<0.001
		vs. female	89	956,002			
	*urban*	male	462	1,396,554	3.22	2.68–3.87	<0.001
		vs. female	149	1,450,139			
	*entire group*	male	817	2,431,240	3.4	2.94–3.93	<0.001
		vs. female	238	2,406,141			
**50–59**	*rural*	male	484	1,084,301	2.47	2.08–2.93	<0.001
		vs. female	180	994,117			
	*urban*	male	664	1,663,738	2.35	2.06–2.68	<0.001
		vs. female	325	1,912,842			
	*entire group*	male	1148	2,748,039	2.4	2.16–2.66	<0.001
		vs. female	505	2,906,959			
**60–69**	*rural*	male	420	667,589	2.01	1.71–2.36	<0.001
		vs. female	226	723,161			
	*urban*	male	614	1,226,637	1.65	1.46–1.86	<0.001
		vs. female	470	1,552,089			
	*entire group*	male	1034	1,894,226	1.78	1.62–1.96	<0.001
		vs. female	696	2,275,250			
**70–79**	*rural*	male	180	350,294	0.81	0.68–0.97	0.019
		vs. female	337	528,443			
	*urban*	male	270	596,787	0.8	0.69–0.93	0.003
		vs. female	539	953,862			
	*entire group*	male	450	947,081	0.8	0.71–0.9	<0.001
		vs. female	876	1,482,305			
**80+**	*rural*	male	70	169,716	0.63	0.48–0.82	<0.001
		vs. female	263	399,864			
	*urban*	male	120	266,716	0.62	0.51–0.76	<0.001
		vs. female	442	606,123			
	*entire group*	male	190	436,432	0.62	0.53–0.73	<0.001
		vs. female	705	1,005,987			

## Data Availability

Data are available upon special request.
